# Safety and tolerability of Rinvecalinase Alfa (DM199) for acute ischemic stroke (ReMEDy1)

**DOI:** 10.1177/17474930251396480

**Published:** 2025-11-06

**Authors:** Bruce CV Campbell, Scott E Kasner, Annette D Lista, John J Volpi, Timothy J Kleinig, Dennis Cordato, Helen M Dewey, Philip MC Choi, Carlos Garcia-Esperon, Ramesh Sahathevan, Tissa Wijeratne, Andrew A Wong, Darshan Ghia, Geoffrey C Cloud, Michael Giuffre, Philip M Bath, for the ReMEDy1 Investigators

**Affiliations:** 1 Department of Medicine and Neurology, Melbourne Brain Centre at the Royal Melbourne Hospital, University of Melbourne, Parkville, VIC, Australia; 2 The Florey Institute of Neuroscience and Mental Health, University of Melbourne, Parkville, VIC, Australia; 3 Department of Neurology, The Royal Melbourne Hospital, Parkville, VIC, Australia; 4 Division of Vascular Neurology, University of Pennsylvania School of Medicine, Philadelphia, PA, USA; 5 DiaMedica Therapeutics Inc., Minnetonka, MN, USA; 6 The Houston Methodist Institute for Academic Medicine, Houston, TX, USA; 7 Department of Neurology, Royal Adelaide Hospital, Adelaide, SA, Australia; 8 Department of Neurology, Liverpool Hospital, Liverpool, NSW, Australia; 9 Department of Neurosciences, Eastern Health and Eastern Health Clinical School, Monash University, Box Hill, VIC, Australia; 10 Department of Neurology, Priority Research Centre for Brain and Mental Health Research, John Hunter Hospital, University of Newcastle, Newcastle, NSW, Australia; 11 Deakin University and St. Vincent’s Hospital Melbourne, Fitzroy, VIC, Australia; 12 Department of Medicine and Neurology, Melbourne Medical School, The University of Melbourne and Western Health, Sunshine Hospital, St Albans, VIC, Australia; 13 Department of Neurology, Royal Brisbane and Women’s Hospital and the University of Queensland, Brisbane, QLD, Australia; 14 Fiona Stanley Hospital, Perth, WA, Australia; 15 Department of Neuroscience, Alfred Hospital, Prahran, Victoria and Department of Neuroscience, School of Translational Medicine, Momash University, Melbourne, VIC, Australia; 16 University of Calgary, Departments of Pediatrics and Cardiology, Libin Cardiovascular Institute, Calgary, AB, Canada; 17 Stroke Trials Unit, Mental Health & Clinical Neuroscience, University of Nottingham, Nottingham, UK

**Keywords:** Ischemic stroke, rinvecalinase alfa, DM199

## Abstract

**Background:**

Rinvecalinase alfa (DM199), a recombinant form of human tissue kallikrein-1 (KLK1), aims to promote local vasodilation to ischemic brain and enhance collateral blood flow. The ReMEDy1 trial tested the safety and tolerability of rinvecalinase alfa in ischemic stroke.

**Methods:**

ReMEDy1 was a phase II, randomized, double-blind, placebo-controlled, study conducted at 13 Australian sites. Ninety-two patients with NIH Stroke Scale (NIHSS) 6–25 were enrolled within 24 h of ischemic stroke onset. Patients were randomized 1:1 to receive rinvecalinase alfa (1 µg/kg intravenous infusion followed by 3 µg/kg subcutaneously every 3 days for 22 days) or placebo. The primary outcome was safety, assessed by adverse events (AEs) and serious adverse events (SAEs). Secondary outcomes included changes in NIHSS, modified Rankin Scale (mRS), and Barthel Index (BI) at Days 22 and 90. Post hoc analyses excluded patients who underwent endovascular therapy (EVT).

**Results:**

The median age was 72, NIHSS 10, and onset-to-randomization was 19.5 h. SAEs were reported in 20/47 (43.5%) rinvecalinase alfa patients and 14/45 (31.1%) placebo patients. Most patients experienced at least one AE; the most common in the rinvecalinase alfa group were constipation (60.9%), oral candidiasis (23.9%), and nausea (17.4%). Stroke-in-evolution by Day 90 occurred in 0 (0%) rinvecalinase alfa patients versus 6 (13.3%) placebo patients; 4/6 (66.7%) placebo patients with stroke-in-evolution died. No significant differences were observed in secondary efficacy outcomes at Day 90. Post hoc analyses in patients not treated with EVT suggested a tendency toward improved excellent global outcomes with rinvecalinase alfa.

**Conclusions:**

Rinvecalinase alfa appeared to be safe and generally well-tolerated in ischemic stroke patients, with potential efficacy in reducing stroke progression. Further studies are needed to confirm efficacy and long-term benefits in patients without EVT.

**Registrations:**

https://www.clinicaltrials.gov/study/NCT03290560

## Graphical abstract

**Graphical abstract:**
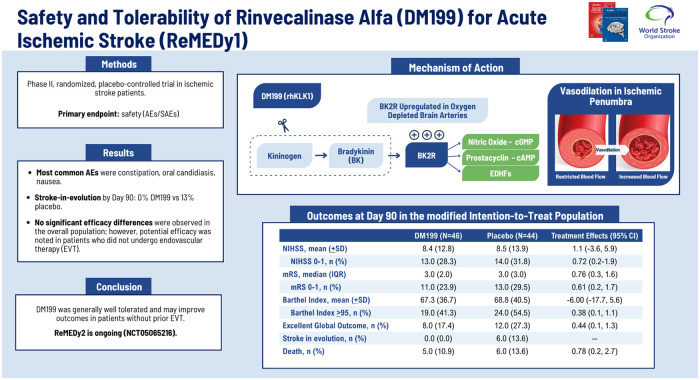

## Introduction

Ischemic stroke remains a leading cause of death and long-term disability worldwide.^1,2^ While intravenous thrombolytics (IVT) and endovascular therapy (EVT) improve outcomes for eligible patients, many do not regain functional independence, highlighting the urgent need for additional treatments. Human tissue kallikrein-1 (KLK1) is an endogenous serine protease primarily produced by the kidneys and is detectable in both blood and urine. KLK1 regulates vasodilation and blood flow by cleaving low-molecular-weight kininogen to release bradykinin. Bradykinin binds to bradykinin 2 receptors which activate nitric oxide, prostacyclin, and endothelial-derived hyperpolarizing factor pathways.^3^ These mediators relax arterial smooth muscle cells, thereby dilating cerebral arteries and enhancing blood flow, particularly to ischemic tissues.^4^

In ischemic stroke, this mechanism may preserve the ischemic penumbra by increasing perfusion to areas of reduced blood flow, preferentially dilating arteries in the penumbra.^4^ In addition, KLK1 may contribute to vascular protection by promoting endothelial repair of unstable plaques and regulating blood pressure, mechanisms that could support post-stroke recovery through vascular recruitment and healing and reducing the risk of recurrent stroke.^5,6^ Lower endogenous KLK1 levels are independently associated with increased risk of both first-ever and recurrent stroke, supporting its hypothesized protective role in cerebrovascular disease.^7,8^

In China, KLK1 derived from human urine (uKLK1), has been an approved and commercially available ischemic stroke treatment for over 15 years with studies suggesting uKLK1 remains effective when commenced up to 48 h after onset.^9,10^ Meta-analyses suggest that uKLK1 therapy improves neurological outcomes and reduces death and dependency three months post-stroke.^10–12^ The preclinical rationale for KLK1 has been detailed in recent reviews.^13–16^ In this phase II trial (ReMEDy1), we evaluated the safety and tolerability of intravenous and subcutaneous administration of recombinant KLK1 (rinvecalinase alfa, DM199) in ischemic stroke.

## Methods

### Study design and population

ReMEDy1 was a randomized, double-blind, placebo-controlled phase II study performed at 13 sites in Australia between February 21, 2018, and January 23, 2020. Patients ⩾ 18 years of age were eligible if they had ischemic stroke with onset ⩽ 24 h and baseline National Institutes of Health Stroke Score (NIHSS) 6–25 (inclusive). Patients could receive IVT, EVT or both, prior to enrollment if NIHSS was 6–25 > 1 h after completion of reperfusion therapy. Patients were excluded from the study if prescribed angiotensin converting enzyme inhibitors (ACEi) and were unable or unwilling to convert to another antihypertensive treatment during the active treatment period (+5 days) of the study, had a past medical history of allergic diathesis (e.g. urticaria, angioedema or anaphylaxis), active or recent serious infection; pregnancy or lack of adequate contraception. A full list of exclusion criteria is available in the Protocol Supplemental Material. One protocol amendment was implemented after the trial began, increasing the sample size from 66 to 100 and adding key exclusion criteria such as a pre-stroke modified Rankin Scale (mRS) score ⩾ 4.

This study was conducted in accordance with the principles of the Declaration of Helsinki and the National Health and Medical Research Council National Statement on Ethical Conduct in Human Research 2007 (updated 2018). The trial was approved by The Royal Melbourne Hospital Human Research Ethics Committee, approval number 2017.294. All patients (or their guardian or legal representative) provided written informed consent. The study was registered on Clinicaltrials.gov (NCT03290560) and endorsed after review by the Australasian Stroke Trials Network.

### Study treatment

**Figure 1.**
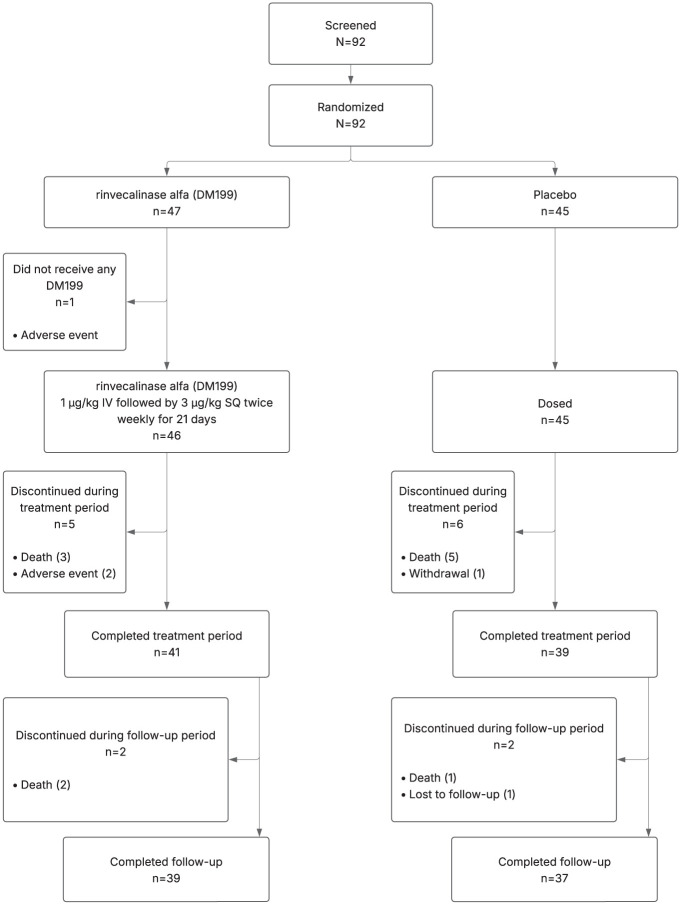
Consort diagram of patients eligible, recruited, numbers followed up and included in analysis.

Patients were randomized to placebo or rinvecalinase alfa, using a central, secure, interactive web response system (IWRS). Individual de-identified patient data, including the randomization details, were generated by Novotech and implemented in IWRS by Clinitec. Randomization was conducted 1:1 without stratification after eligibility was confirmed. The random allocation sequence was implemented using the IWRS, which concealed treatment assignments until interventions were allocated. Both the participant and investigators were blinded to the treatment assignment, except for a designated unblinded pharmacist responsible for preparing and dispensing the assigned study treatment. An initial, single 40-minute intravenous (IV) infusion of placebo (saline) or rinvecalinase alfa 1 µg/kg was delivered with polyolefin infusion materials, followed by subcutaneous (SC) injections of placebo or rinvecalinase alfa 3 µg/kg starting 2–12 h after the IV infusion and continuing every 72 ± 2 h for the remainder of the 22-day treatment period (total eight SC doses) (Figure 1). Treatment had to commence within 24 ± 2 h after stroke onset. Study drug accountability was closely monitored throughout the trial, with all used and unused product reconciled, and final disposition records, including destruction certificates, maintained in the site file and provided to the sponsor.

### Outcomes

The primary outcome was safety, assessed by incidence of adverse events (AEs) and serious adverse events (SAEs) based on physical examination, vital signs, 12-lead electrocardiogram (ECG), and clinical laboratory measurements (hematology, serum chemistry, urinalysis). Treatment-emergent AEs (TEAEs) were defined as AEs with an onset after administration of the study treatment, or when a pre-existing medical condition increases in severity or frequency after study treatment administration. Adverse events and other symptoms were graded according to National Cancer Institute’s Common Toxicity Criteria for Adverse Events version 4.03 (CTCAE v4.03). Tolerability was assessed by incidence and severity of injection site adverse events graded according to CTCAE v4.03 Grading of Local (Injection Site) Adverse Event Intensity.

Secondary outcomes included efficacy as assessed by change in NIHSS, Barthel index (BI), mRS from baseline on Days 22 and 90. Evaluation of pharmacokinetic findings were assessed at baseline and on Days 2, 22, and 90. Clinical outcomes were defined as excellent (NIHSS ⩽ 1) and/or (mRS ⩽ 1) and/or excellent global outcomes (NIHSS ⩽ 1, mRS ⩽ 1, and BI ⩾ 95) at Day 90. Stroke-in-evolution was defined as neurological symptoms that continued to worsen or progress over time per investigator judgment.

### Pharmacokinetic assessments

Frozen plasma samples collected during the clinical study were analyzed to evaluate the concentration of KLK1 in human K_2_EDTA plasma using a validated electrochemiluminescent immunoassay. This assay does not distinguish between endogenous KLK1 and recombinant KLK1 (rinvecalinase alfa).

### Statistical analysis

No formal sample size estimation was performed. The target enrollment size of 100 was considered adequate to evaluate the safety and tolerability of rinvecalinase alfa based on previous clinical studies of rinvecalinase alfa and uKLK1.^17^ Analyses were conducted in the safety population, which included all randomized patients who received at least one dose of study treatment, and the modified intention-to-treat (mITT) population, which included all randomized patients who received at least one dose of study treatment and had at least one post-dose efficacy assessment. Missing data were not imputed, and analyses included all available data up to the point of discontinuation for subjects who withdrew prior to study completion. Analyses used binary logistic regression (BLR) for dichotomous data, ordinal logistic regression (OLR) for ordered categorical data and multiple linear regression (MLR). Analyses of NIHSS, mRS, Barthel Index, and composite Excellent Outcome/Excellent Global Outcome were adjusted for baseline age and NIHSS score (both continuous). Safety-related outcomes were not adjusted. No adjustments were made for multiplicity. Post hoc analyses were conducted to examine treatment effects in patients who did or did not receive endovascular therapy (EVT).

## Results

### Study population

Of 92 patients, 47 (51%) were randomized to rinvecalinase alfa, and 45 (49%) to placebo (Figure 1). Median (IQR) age was 72 (60–81) years, and 58.2% were male. The median baseline NIHSS score was 10 (8–15) and median time from symptom onset to randomization was 19.5 (15.3–23.6) h. Baseline characteristics were comparable between treatment groups except fewer patients in the rinvecalinase alfa group received combined pre-treatment with IVT and EVT (5 [10.9%] versus placebo 12 [26.7%]; Table 1).

**Table 1.** Baseline characteristics in the safety population.

	Rinvecalinase alfa (N = 46)	Placebo (N = 45)
Age (years), median (IQR)	73 (60.0–80.0)	72 (66.0–83.0)
Sex, male, n (%)	25 (54.3)	28 (62.2)
Race, n (%)		
White	41 (89.1)	40 (88.9)
Asian	5 (10.9)	4 (8.9)
Native Hawaiian or Other Pacific Islander	0 (0.0)	1 (2.2)
Weight (kg), median (IQR)	80.5 (68.0–89.6)	85 (72.0–95.0)
Height (m), median (IQR)	1.7 (1.6–1.8)	1.7 (1.6–1.8)
Body mass index (kg/m^2^), median (IQR)	28.2 (23.7–31.5)	29.3 (25.7–32.9)
Medical history, n (%)		
Atrial fibrillation	12 (26.1)	19 (42.2)
Hypercholesterolemia	14 (30.4)	14 (31.1)
Hypertension	32 (69.6)	32 (71.1)
Myocardial ischemia	7 (15.2)	10 (22.2)
Diabetes mellitus	15 (32.6)	13 (28.9)
Chronic kidney disease	2 (4.3)	2 (4.4)
Smoker, current	4 (8.7)	3 (6.7)
NIHSS, median (IQR)^a^	10 (8.0–14.0)	9.5 (7.5–17.0)
mRS, median (IQR)^a^	0 (0.0–0.0)	0 (0.0–0.5)
Barthel index, median (IQR)^a^	100 (100.0–100.0)	100 (100.0–100.0)
Systolic blood pressure (mmHg), median (IQR)	133.5 (124.0–146.0)	135 (122.0–156.0)
Time, onset to randomization (h), median (IQR)	18.7 (14.8–22.9)	21 (15.8–24.8)
Reperfusion therapy, n (%)		
Thrombolysis only	13 (28.3)	8 (17.8)
Endovascular therapy only	16 (34.8)	12 (26.7)
Both thrombolysis and endovascular therapy	5 (10.9)	12 (26.7)
None	12 (26.1)	13 (28.9)

a Modified intention-to-treat population.

### Safety outcomes

Serious adverse events (SAEs) were reported in 20 (43.5%) patients receiving rinvecalinase alfa and 14 (31.1%) patients receiving placebo. Most patients experienced at least one treatment-emergent adverse event (TEAE) during the study (Table 2). The most common events in the rinvecalinase alfa group were constipation (60.9%), oral candidiasis (23.9%), and nausea (17.4%). Stroke-in-evolution occurred in 0 patients in the rinvecalinase alfa group versus 6 (13.3%) with placebo. Hemorrhagic transformation occurred in 3 (6.6%) in the rinvecalinase alfa group and 0 with placebo. Treatment-emergent related SAEs included bradycardia, increased transaminases, and flushing, each occurring in 1 (2.2%) patient in the rinvecalinase alfa group and 0 with placebo. Although no adverse events of special interest were prospectively defined in the protocol, events potentially relevant to the mechanism of action of rinvecalinase alfa (e.g. hypotension, angioedema) were specifically evaluated. Hypotension occurred in 1 (2.2%) patient in the rinvecalinase alfa group and 5 (11.1%) with placebo. Angioedema occurred in 1 (2.2%) patient receiving rinvecalinase alfa and 0 receiving placebo. In the safety population, there were five deaths in the rinvecalinase alfa group and six with placebo; none were considered related to study drug.

**Table 2.** Summary of adverse events in safety population.

	Rinvecalinase alfa (N = 46)	Placebo (N = 45)
Adverse events, n (%)	46 (100.0)	43 (95.6)
Treatment-related	12 (26.1)	6 (13.3)
Treatment-related serious	3 (6.5)	0 (0.0)
Leading to study drug discontinuation	4 (8.7)	3 (6.7)
Treatment-emergent adverse events (⩾10%), n (%)		
Gastrointestinal disorders	34 (73.9)	22 (48.9)
Constipation	28 (60.9)	14 (31.1)
Nausea	9 (19.6)	4 (8.9)
Infections	23 (50.0)	16 (35.6)
Oral candidiasis	11 (23.9)	8 (17.8)
Urinary tract infection	8 (17.4)	8 (17.8)
Pneumonia	2 (4.3)	5 (11.1)
Nervous system disorders	17 (37.0)	22 (48.9)
Headache	7 (15.2)	5 (11.1)
Administration site conditions	19 (41.3)	11 (24.4)
Pyrexia	6 (13.0)	2 (4.4)
Pain	5 (10.9)	2 (4.4)
Metabolism and nutrition disorders	15 (32.6)	12 (26.7)
Hypokalemia	5 (10.9)	2 (4.4)
Investigations	14 (30.4)	10 (22.2)
Weight decreased	5 (10.9)	2 (4.4)
Injury, poisoning, and procedural complications	11 (23.9)	12 (26.7)
Contusion	6 (13.0)	4 (8.9)
Fall	2 (4.3)	5 (11.1)
Cardiac disorders	11 (23.9)	11 (24.4)
Atrial fibrillation	7 (15.2)	7 (15.6)
Musculoskeletal and connective tissue disorders	10 (21.7)	12 (26.7)
Musculoskeletal pain	2 (4.3)	5 (11.1)
Arthralgia	1 (2.2)	5 (11.1)
Vascular disorders	10 (21.7)	11 (24.4)
Hypotension	1 (2.2)	5 (11.1)
Respiratory, thoracic, and mediastinal disorders	14 (30.4)	6 (13.3)
Pneumonia aspiration	4 (8.7)	5 (11.1)
Psychiatric disorders	12 (26.1)	7 (15.6)
Depression	5 (10.9)	3 (6.7)
Skin and subcutaneous tissue disorders	10 (21.7)	4 (8.9)
Rash	6 (13.0)	2 (4.4)
Serious adverse events, n (%)	20 (43.5)	14 (31.1)
Stroke-in-evolution	0 (0.0)	6 (13.3)
Haemorrhagic transformation	3 (6.5)	0 (0.0)
Treatment-emergent related serious adverse events, n (%)		
Cardiac disorders	1 (2.2)	0 (0.0)
Bradycardia	1 (2.2)	0 (0.0)
Investigations	1 (2.2)	0 (0.0)
Transaminases increased	1 (2.2)	0 (0.0)
Vascular disorders	1 (2.2)	0 (0.0)
Flushing	1 (2.2)	0 (0.0)

### Efficacy outcomes

**Figure 2.**
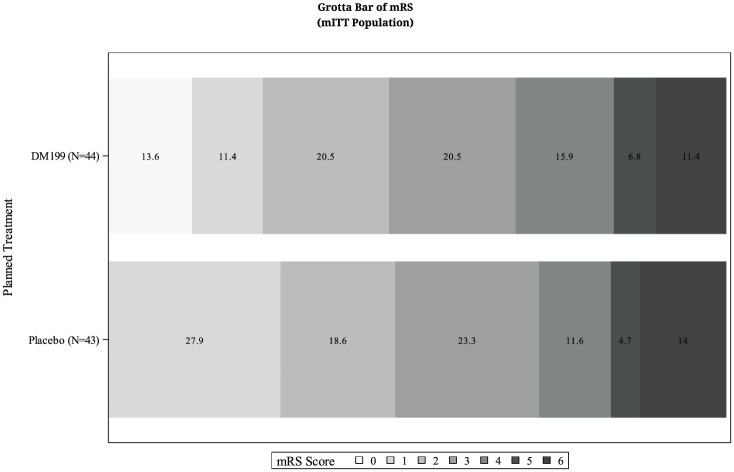
Modified rankin scale outcome at day 90 in the modified intention-to-treat population.^a^ ^a^Data reflect modified Rankin Scale (mRS) scores at Day 90. Three patients (1 lost to follow-up after Day 79, 2 discontinued due to adverse events) were excluded.

No differences were observed in clinical outcomes, whether defined as excellent ((NIHSS ⩽ 1) and/or (mRS ⩽ 1)) or “excellent global” outcomes ((NIHSS ⩽ 1, mRS ⩽ 1, and BI ⩾ 95) at Day 90) (Table 3). Figure 2 shows the mRS distribution at Day 90. Post hoc analyses are reported in the Supplemental Material (Figure S1, Tables S1–S6).

**Table 3.** Outcomes at day 90 in the modified intention-to-treat population.

	Rinvecalinase alfa (N = 46)	Placebo (N = 44)	Treatment effects (95% CI)
NIHSS, mean (±SD)^a^	8.4 (12.81)	8.5 (13.93)	1.1 (−3.68, 5.90)
NIHSS 0–1, n (%)	13.0 (28.30)	14.0 (31.80)	0.72 (0.28, 1.97)
mRS, median (IQR)^b^	3.0 (2.00)	3.0 (3.00)	0.76 (0.36, 1.61)
mRS 0–1, n (%)	11.0 (23.90)	13.0 (29.50)	0.61 (0.21, 1.75)
Barthel Index, mean (±SD)^a^	67.3 (36.78)	68.8 (40.52)	−6.00 (−17.72, 5.66)
Barthel Index ⩾ 95, n (%)	19.0 (41.30)	24.0 (54.50)	0.38 (0.13, 1.11)
Excellent global outcome, n (%)^c^	8.0 (17.40)	12.0 (27.30)	0.44 (0.14, 1.35)
Stroke in evolution, n (%)^d^	0.0 (0.00)	6.0 (13.60)	–
Death, n (%)	5.0 (10.9)	6.0 (13.60)	0.78 (0.22, 2.74)

CI = confidence interval; SD = standard deviation; IQR = interquartile range.

a NIHSS, mean (±SD) and Barthel Index, mean (±SD) treatment effects are adjusted mean treatment difference (from multiple linear regression).

b mRS, median (IQR) treatment effect is adjusted common odds ratio from ordinal logistic regression.

For all other results, treatment effect is given as adjusted odds ratio from binary logistic regression model.

c Excellent global outcomes were defined as NIHSS ⩽ 1, mRS ⩽ 1, and BI ⩾ 95.

d The odds ratio for stroke in evolution is not estimable due to zero events in the rinvecalinase alfa treatment group.

### Pharmacokinetics

There was significantly increased plasma KLK1 exposure at Days 2 and 22 in patients randomized to rinvecalinase alfa compared to placebo; both rinvecalinase alfa and placebo groups had low levels on Day 90 (Table 4).

**Table 4.** Plasma KLK1 concentrations.

Study day	Rinvecalinase alfa	Placebo	Adjusted mean difference (LS means)^a^
Day 2, N	43	42	
[KLK1] (ng/mL), mean (±SD)	2.78 (2.15)	0.55 (0.90)	2.18 (1.57, 2.79)
Day 22, N	40	39	
[KLK1] (ng/mL), mean (±SD)	3.67 (2.31)	0.31 (0.59)	3.16 (2.53, 3.79)
Day 90, N	38	33	
[KLK1] (ng/mL), mean (±SD)	0.47 (1.04)	0.50 (0.76)	−0.05 (−0.70, 0.60)

LS = least squares mean; SD = standard deviation.

a LS Means are from a mixed model for repeated measures (MMRM) with fixed effects of baseline KLK1, treatment, visit, and treatment*visit interaction and random effect of subject.

## Discussion

The ReMEDy1 trial findings confirm the safety, tolerability, and feasibility of rinvecalinase alfa in patients with ischemic stroke. No differences in SAEs or AEs were observed between rinvecalinase alfa and placebo. No significant differences were noted in clinical outcomes, including NIHSS, mRS, and BI scores, across treatment arms. However, rinvecalinase alfa was associated with a reduction in the absolute risk of stroke-in-evolution by 13.6%, and a corresponding decrease in associated mortality.

The observed reduction in stroke-in-evolution suggests that this rinvecalinase alfa dosing regimen offering extended KLK1 supplementation may help protect the penumbra from subacute ischemic damage by increasing regional blood flow. These findings align with results from previous trials investigating uKLK1. In this population, we observed low KLK1 levels in the weeks after AIS, and this persisted for 3 weeks in the placebo group. Low KLK1 levels have been independently linked to an increased incidence of ischemic stroke, poorer outcomes, and a higher risk of recurrent stroke.^10,17^ Similarly, treatment with uKLK1 has demonstrated improved outcomes in ischemic stroke patients and a reduced risk of stroke recurrence.^10,18^ Given that the highest risk for recurrent stroke occurs within the first 90 days post-stroke, future studies exploring whether chronic administration of rinvecalinase alfa can reduce long-term stroke recurrence are warranted.^18^

ReMEDy1 was designed to evaluate rinvecalinase alfa in a diverse patient population, including those undergoing reperfusion therapy, to guide future clinical research. An unexpectedly high proportion of patients (49.5%) received EVT, exceeding typical rates of <10%.^19–21^ Prior randomized trials of human urinary kallikrein in China largely excluded EVT-pretreated patients.^22–24^ In ReMEDy1, patients pretreated with EVT did not benefit from rinvecalinase alfa. In contrast, exploratory analyses of patients who did not undergo EVT suggested a potential treatment signal with rinvecalinase alfa in excellent global outcomes, and fewer experienced stroke-in-evolution or death compared with placebo. While underpowered for definitive conclusions, these results align with prior non-EVT studies of kallikrein in China. The higher frequency of EVT in ReMEDy1, as well as an imbalance in EVT/IVT pretreatment (10.9% in the rinvecalinase alfa group versus 26.7% with placebo), may have masked the ability to observe a treatment effect in the overall population. Although exploratory, an interaction between rinvecalinase and EVT treatment status was observed for mRS 0−1.

Rinvecalinase alfa was administered as a single 1-µg/kg IV dose, followed by 3-µg/kg SC injections every 3 days over a 22-day period. This regimen offers a similar treatment period as the 7- to 21-day daily IV infusion schedule used for uKLK1. Preclinical data demonstrated that bradykinin B2 receptor expression remains elevated for up to 7 days post-reperfusion, supporting the rationale for extended dosing.^25^ Pharmacokinetic studies confirmed that rinvecalinase alfa achieves plasma concentrations comparable to the standard IV dose of uKLK1 used in China for ischemic stroke treatment.^26,27^ In ReMEDy1, the IV dose was 1 µg/kg using polyolefin materials. However, subsequent compatibility testing revealed significant adsorption to polyolefin, resulting in a delivered dose of approximately 0.5 µg/kg. The ongoing ReMEDy2 trial delivers a 0.5 µg/kg IV dose using polyvinyl chloride infusion materials, which are non-adsorptive.^28^

This trial had several strengths. It was a double-blind, randomized study conducted in a moderate to severe stroke population. The study design was informed by prior clinical data on uKLK1 for ischemic stroke, and rinvecalinase alfa was dosed at levels comparable to uKLK1.

Limitations include heterogeneity in reperfusion therapies as 72.5% of patients received IVT and/or EVT before rinvecalinase alfa. While this diversity enhanced generalizability regarding safety in a moderate to severe stroke population, it confounded the assessment of functional outcomes. In addition, the study was conducted across multiple centers with potentially varying clinical practices and patient populations. The trial was small, conducted in a single country and not powered to evaluate efficacy. The timing of the qualifying NIHSS, which occurred >1 h after IVT or EVT, was a compromise to balance excluding patients with an excellent immediate response to reperfusion while avoiding excessive delays in initiating rinvecalinase alfa treatment. However, we acknowledge that accurate neurological assessment during this window can be limited by procedural sedation, intubation, or other post-reperfusion factors, which may have influenced baseline NIHSS scores and the selection of enrolled patients. We did not perform post-treatment perfusion imaging. Future studies will be needed to assess mechanistic hypotheses and the theoretical concern of steal phenomenon with vasodilatory therapies.

## Conclusions

In summary, rinvecalinase alfa demonstrated a favorable safety and tolerability profile in this trial. While no clear improvements in overall stroke outcomes were observed compared to placebo, numerical reductions in stroke-in-evolution and severe recurrence were noted. In exploratory post hoc analyses, patients not pretreated with EVT showed a tendency toward improved functional outcomes with rinvecalinase alfa, whereas no additional benefit was seen in EVT-pretreated patients, likely reflecting the strong therapeutic effects of EVT. These exploratory findings suggest that rinvecalinase alfa may have the greatest therapeutic potential in patients who do not undergo EVT, a population in which restoration of collateral circulation remains an unmet need. A Phase II/III study (ReMEDy2) is currently underway to evaluate rinvecalinase alfa in ischemic stroke patients, specifically excluding those with large-vessel occlusions eligible for EVT (ClinicalTrials.gov Identifier: NCT05065216).

## Supplemental Material

sj-pdf-1-wso-10.1177_17474930251396480 – Supplemental material for Safety and tolerability of Rinvecalinase Alfa (DM199) for acute ischemic stroke (ReMEDy1) Supplemental material, sj-pdf-1-wso-10.1177_17474930251396480 for Safety and tolerability of Rinvecalinase Alfa (DM199) for acute ischemic stroke (ReMEDy1) by Bruce CV Campbell, Scott E Kasner, Annette D Lista, John J Volpi, Timothy J Kleinig, Dennis Cordato, Helen M Dewey, Philip MC Choi, Carlos Garcia-Esperon, Ramesh Sahathevan, Tissa Wijeratne, Andrew A Wong, Darshan Ghia, Geoffrey C Cloud, Michael Giuffre and Philip M Bath in International Journal of Stroke
