# Supplementary material for: Safety and tolerability of Rinvecalinase Alfa (DM199) for acute ischemic stroke (ReMEDy1)
Source: Int J Stroke. 2025 Nov 6;21(6):861–70. doi: 10.1177/17474930251396480 (PMC13291399; doi:10.1177/17474930251396480)
Supplement: sj-pdf-1-wso-10.1177_17474930251396480 – Supplemental material for Safety and tolerability of Rinvecalinase Alfa (DM199) for acute ischemic stroke (ReMEDy1) [file sj-pdf-1-wso-10.1177_17474930251396480.pdf]

Supplementary Appendix

Table of contents:

Supplemental Results 2

Figure S1: modified Rankin Scale Outcome at Day 90 in Patients with or without Endovascular Therapy 3

Table S1: Baseline Characteristics in the Safety Population in Patients with no Endovascular Therapy 4

Table S2: Outcomes at Day 90 in Patients with No Endovascular Therapy 6

Table S3: Baseline Characteristics in the Safety Population in Patients with Endovascular Therapy 7

Table S4: Outcomes at Day 90 in Patients with Endovascular Therapy 9

Table S5: Post Hoc Interaction Analyses Between Treatment and Reperfusion Therapy (EVT ± IVT) 10

Table S6: Summary of All Serious Adverse Events 11

List of ReMEDy1 Sites, Investigators, Study Coordinators, and Research Nurses 13

Supplemental results:

Post hoc subgroup analyses in patients who did not receive endovascular therapy

Among the overall patient population, 46 patients were not pretreated with EVT (25 patients who received rinvecalinase alfa and 21 patients who received placebo). Baseline characteristics were generally similar between treatment groups (**Table S1**). **Figure S1A** shows the mRS distribution at Day 90. Efficacy outcomes in this subgroup are reported in **Table S2**.

Post hoc subgroup analyses in patients who received endovascular therapy

Among the overall patient population, 45 patients were pretreated with EVT (21 patients who received rinvecalinase alfa and 24 patients who received placebo). Baseline characteristics were generally similar between treatment groups (**Table S3**). **Figure S1B** shows the mRS distribution at Day 90. Efficacy outcomes in this subgroup are reported in **Table S4**. Exploratory interaction analyses between treatment assignment and EVT status were conducted and are summarized in **Table S5.**

Figure S1 A) modified Rankin Scale Outcome at Day 90 in Patients with No Endovascular Therapy

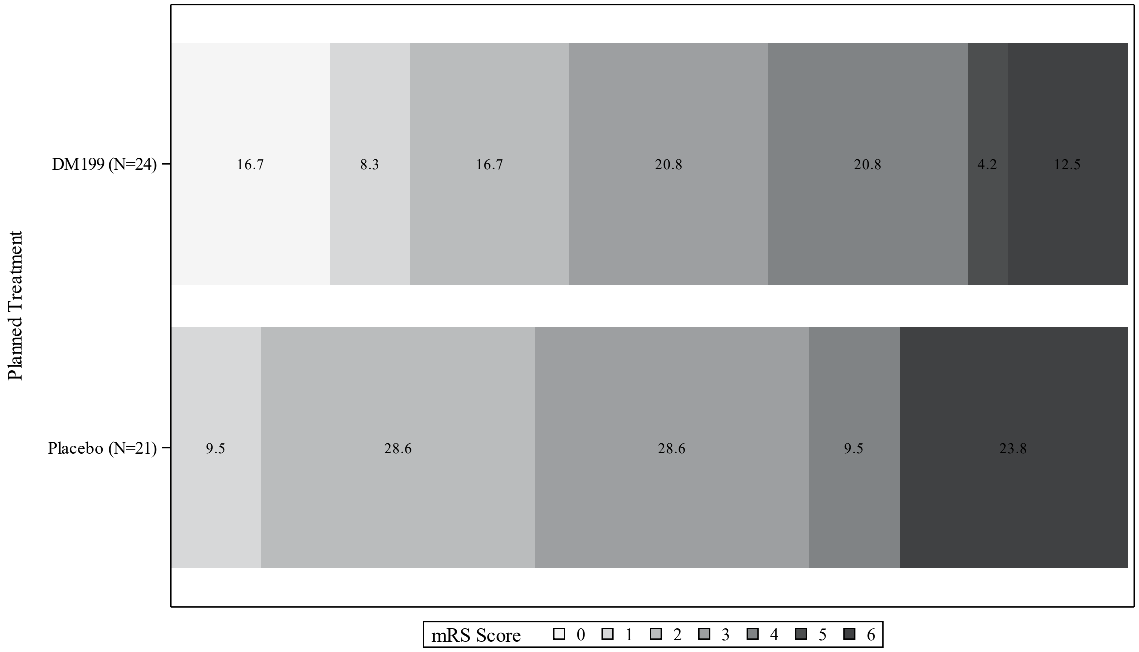

B) modified Rankin Scale Outcome at Day 90 in Patients with Endovascular Therapy

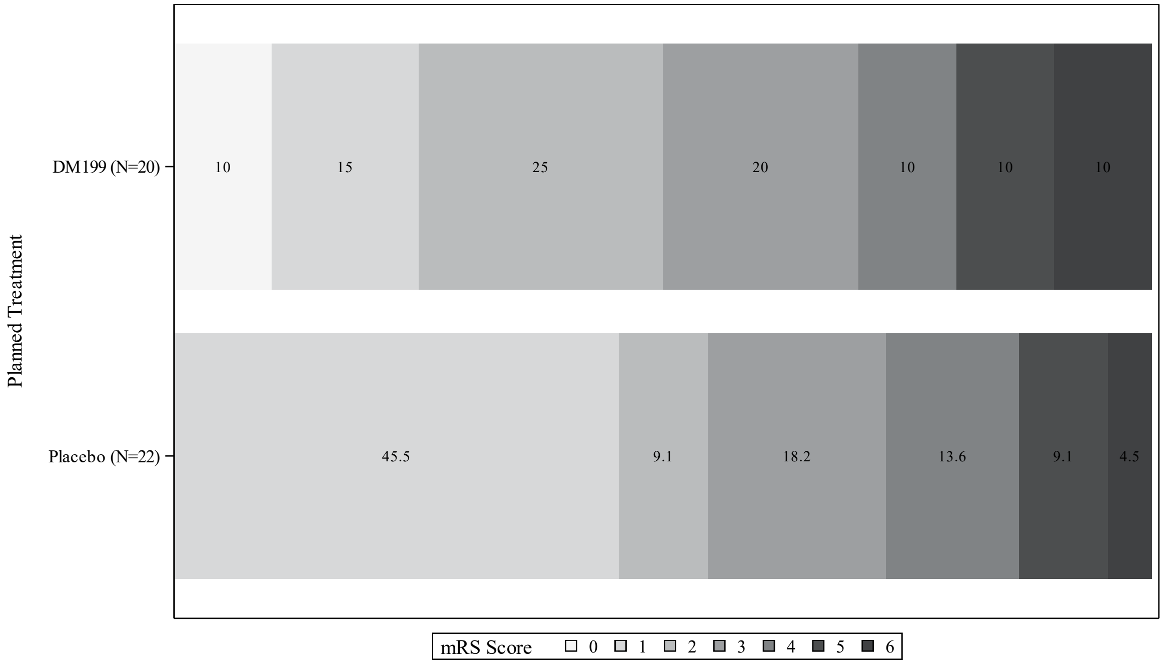

A score of ≤1 on the modified Rankin scale is considered to indicate a favorable outcome.

Table S1. Baseline Characteristics in the Safety Population in Patients with no Endovascular Therapy

	**Rinvecalinase alfa**<br><br>(N=25)	**Placebo**<br><br>(N=21)
Age (years), median (IQR)	69 (60.0-77.0)	71 (67.0-85.0)
Sex, male, n (%)	17 (68.0)	13 (61.9)
Race, n (%)		
White	21 (84.0)	18 (85.7)
Asian	4 (16.0)	3 (14.3)
Native Hawaiian or Other Pacific Islander	0 (0.0)	0 (0.0)
Weight (kg), median (IQR)	84.6 (73.0-93.3)	80.9 (70.0-91.0)
Height (m), median (IQR)	1.8 (1.7-1.8)	1.7 (1.6-1.8)
Body mass index (kg/m^2), median (IQR)	27.8 (25.3-30.4)	26.8 (25.2-31.2)
Medical history, n (%)		
Atrial fibrillation	4 (16.0)	8 (38.1)
Hypercholesterolemia	10 (40.0)	9 (42.9)
Hypertension	19 (76.0)	19 (90.5)
Myocardial ischemia	4 (16.0)	6 (28.6)
Diabetes mellitus	12 (48.0)	10 (47.6)
Chronic kidney disease	1 (4.0)	1 (4.8)
Smoker, current	4 (16.0)	1 (4.8)

NIHSS, median (IQR)[1]	9 (7.0-14.0)	9 (8.0-16.0)
mRS, median (IQR)[1]	0 (0.0-0.0)	0 (0.0-2.0)
Barthel index, median (IQR)[1]	100 (100.0-100.0)	100 (95.0-100.0)
Systolic blood pressure (mmHg), median (IQR)	133 (124.0-146.0)	142 (126.0-159.0)
Time, onset to randomization (hr), median (IQR)	18.3 (16.3-22.3)	24.1 (18.4-25.5)
Reperfusion therapy, n (%)		
Thrombolysis only	13 (52.0)	8 (38.1)
None	9 (36.0)	13 (61.9)

[1]modified Intention-to-Treat Population

Table S2. Outcomes at Day 90 in Patients with No Endovascular Therapy

	**Rinvecalinase alfa**<br><br>(N=25)	**Placebo**<br><br>(N=21)	**Treatment Effects**<br><br>(95% CI)
NIHSS, mean (±SD)[2]	8.9 (13.38)	12.4 (17.15)	-0.60 (-7.91, 6.65)
NIHSS 0-1, n (%)	9.0 (36.00)	3.0 (14.30)	3.80 (0.72, 20.19)
mRS, median (IQR)[3]	3.0 (2.00)	3.0 (2.00)	1.18 (0.39, 3.69)
mRS 0-1, n (%)	6.0 (24.00)	2.0 (9.50)	6.12 (0.33, 114.43)
Barthel Index, mean (±SD)[2]	65.0 (37.91)	64.0 (43.38)	-8.20 (-24.12, 7.74)
Barthel Index ≥95, n (%)	9.0 (36.00)	10.0 (47.60)	0.32 (0.06, 1.64)
Excellent Global Outcome, n (%)[4]	5.0 (20.00)	1.0 (4.80)	16.99 (0.37, 785.01)
Stroke in evolution, n (%)[5]	0.0 (0.00)	4.0 (19.0)	--
Death, n (%)	3.0 (12.00)	5.0 (23.80)	0.44 (0.09, 2.10)

CI = confidence interval; SD = standard deviation; IQR = interquartile range

[2]NIHSS, mean (±SD) and Barthel Index, mean (±SD) treatment effects are adjusted mean treatment difference (from multiple linear regression).

[3]mRS, median (IQR) treatment effect is adjusted common odds ratio from ordinal logistic regression.

For all other results, treatment effect given as adjusted odds ratio from binary logistic regression model.

[4]Excellent global outcomes were defined as NIHSS ≤1, mRS ≤1, and BI ≥95.

[5]The odds ratio for stroke in evolution is not estimable due to zero events in the rinvecalinase alfa treatment group.

Table S3. Baseline Characteristics in the Safety Population in Patients with Endovascular Therapy

	Rinvecalinase alfa (N=21)	Placebo (N=24)
Age (years), median (IQR)	76 (66.0-81.0)	72 (65.5-79.5)
Sex, male, n (%)	8 (38.1)	15 (62.5)
Race, n (%)		
White	20 (95.2)	22 (91.7)
Asian	1 (4.8)	1 (4.2)
Native Hawaiian or Other Pacific Islander	0 (0.0)	1 (4.2)
Weight (kg), median (IQR)	78.4 (67.0-85.0)	89.1 (72.5-97.2)
Height (m), median (IQR)	1.6 (1.6-1.7)	1.7 (1.6-1.8)
Body mass index (kg/m^2), median (IQR)	29.3 (23.2-31.6)	30.9 (27.0-33.4)
Medical history, n (%)		
Atrial fibrillation	8 (38.1)	11 (45.8)
Hypercholesterolemia	4 (19.0)	5 (20.8)
Hypertension	13 (61.9)	13 (54.2)
Myocardial ischemia	3 (14.3)	4 (16.7)
Diabetes mellitus	3 (14.3)	3 (12.5)
Chronic kidney disease	1 (4.8)	1 (4.2)
Smoker, current	0 (0.0)	2 (8.3)
NIHSS, median (IQR)[1]	12 (9.0-14.0)	11 (7.0-18.0)

mRS, median (IQR)[1]	0 (0.0-0.0)	0 (0.0-0.0)
Barthel index, median (IQR)[1]	100 (100.0-100.0)	100 (100.0-100.0)
Systolic blood pressure (mmHg), median (IQR)	134 (124.0-141.0)	134 (114.5-143.5)
Time, onset to randomization (hr), median (IQR)	19.1 (14.8-22.9)	18.9 (14.3-22.6)
Reperfusion therapy, n (%)		
Endovascular therapy only	16 (76.2)	12 (50.0)
Both thrombolysis and endovascular therapy	5 (23.8)	12 (50.0)

[1]modified Intention-to-Treat Population

Table S4. Outcomes at Day 90 in Patients with Endovascular Therapy

	**Rinvecalinase alfa** (N=21)	**Placebo** (N=23)	**Treatment Effects** (95% CI)
NIHSS, mean (±SD)[2]	7.8 (12.40)	5.0 (9.20)	2.90 (-3.04, 8.94)
NIHSS 0-1, n (%)	4.0 (19.00)	11.0 (47.80)	0.22 (0.05, 0.93)
mRS, median (IQR)[3]	3.0 (2.00, 4.00)	2.0 (1.00, 4.00)	0.50 (0.17, 1.47)
mRS 0-1, n (%)	5.0 (23.80)	11.0 (47.80)	0.29 (0.07, 1.17)
Barthel Index, mean (±SD)[2]	70.0 (36.12)	73.0 (38.19)	-3.60 (-21.13, 14.01)
Barthel Index ≥95, n (%)	10.0 (47.60)	14.0 (60.90)	0.66 (0.13, 3.28)
Excellent Global Outcome, n (%)[4]	3.0 (14.30)	11.0 (47.80)	0.14 (0.03, 0.70)
Stroke in evolution, n (%)[5]	0.0 (0.00)	2.0 (8.70)	--
Death, n (%)	2.0 (9.50)	1.0 (4.30)	2.32 (0.19, 27.59)

CI = confidence interval; SD = standard deviation; IQR = interquartile range

[2]NIHSS, mean (±SD) and Barthel Index, mean (±SD) treatment effects are adjusted mean treatment difference (from multiple linear regression).

[3]mRS, median (IQR) treatment effect is adjusted common odds ratio from ordinal logistic regression.

For all other results, treatment effect given as adjusted odds ratio from binary logistic regression model.

[4]Excellent global outcomes were defined as NIHSS ≤1, mRS ≤1, and BI ≥95.

[5]The odds ratio for stroke in evolution is not estimable due to zero events in the rinvecalinase alfa treatment group.

Table S5. Post Hoc Interaction Analyses Between Treatment and Reperfusion Therapy (EVT ± IVT)

Outcome	Model Type	Covariates	Interaction Term (Treatment × EVT/IVT)	p-value	Interpretation
mRS 0–1	Binary logistic	Age, baseline NIHSS, treatment, EVT, treatment x EVT interaction	Significant	0.0397	Suggest treatment effect may differ by EVT status
mRS 0–1	Binary logistic	Age, NIHSS, treatment, EVT/IVT, treatment x EVT/IVT interaction	Not significant	0.0735	No clear evidence of interaction
mRS (ordinal)	Ordinal logistic	Age, NIHSS, treatment, EVT, treatment x EVT interaction	Not significant	0.1810	No clear evidence of interaction
mRS (ordinal)	Ordinal logistic	Age, NIHSS, treatment, EVT/IVT, treatment x EVT/IVT interaction	Not significant	0.2747	No clear evidence of interaction

Table S6. Summary of All Serious Adverse Events

	**Rinvecalinase alfa** (N=46)	**Placebo** (N=45)	**All** (N=91)
Nervous system disorders	6 (13.0)	11 (24.4)	17 (18.7)
Stroke in evolution	0	6 (13.3)	6 (6.6)
Cerebral infarction	1 (2.2)	1 (2.2)	2 (2.2)
Carotid artery thrombosis	0	1 (2.2)	1 (1.1)
Cerebral artery occlusion	1 (2.2)	0	1 (1.1)
Cerebrovascular accident	1 (2.2)	0	1 (1.1)
Cognitive disorder	1 (2.2)	0	1 (1.1)
Hemorrhagic transformation stroke	1 (2.2)	0	1 (1.1)
Seizure	0	1 (2.2)	1 (1.1)
Sensory disturbance	1 (2.2)	0	1 (1.1)
Syncope	0	1 (2.2)	1 (1.1)
Transient ischemic attack	0	1 (2.2)	1 (1.1)
Infections and infestations	4 (8.7)	3 (6.7)	7 (7.7)
Bacterial sepsis	0	1 (2.2)	1 (1.1)
Lower respiratory tract infection	1 (2.2)	0	1 (1.1)
Pelvic inflammatory disease	1 (2.2)	0	1 (1.1)
Pneumonia	1 (2.2)	0	1 (1.1)
Sepsis	0	1 (2.2)	1 (1.1)
Streptococcal bacteremia	1 (2.2)	0	1 (1.1)
Tuberculosis	0	1 (2.2)	1 (1.1)
Cardiac disorders	1 (2.2)	2 (4.4)	3 (3.3)
Acute myocardial infarction	0	2 (4.4)	2 (2.2)
Bradycardia	1 (2.2)	0	1 (1.1)
Respiratory, thoracic, and mediastinal disorders	3 (6.5)	0	3 (3.3)
Acute pulmonary oedema	1 (2.2)	0	1 (1.1)
Pneumonia aspiration	1 (2.2)	0	1 (1.1)
Respiratory failure	1 (2.2)	0	1 (1.1)
Eye disorders	1 (2.2)	1 (2.2)	2 (2.2)
Retinal artery occlusion	1 (2.2)	1 (2.2)	2 (2.2)
Gastrointestinal disorders	1 (2.2)	1 (2.2)	2 (2.2)
Colitis ischemic	1 (2.2)	0	1 (1.1)
Gastrointestinal hemorrhage	0	1 (2.2)	1 (1.1)
Investigations	2 (4.3)	0	2 (2.2)
Oxygen saturation decreased	1 (2.2)	0	1 (1.1)
Transaminases increased	1 (2.2)	0	1 (1.1)
Renal and urinary disorders	2 (4.3)	0	2 (2.2)
Acute kidney injury	2 (4.3)	0	2 (2.2)
Vascular disorders	2 (4.3)	0	2 (2.2)

Flushing	1 (2.2)	0	1 (1.1)
Hypertension	1 (2.2)	0	1 (1.1)
General disorders and administration site conditions	0	1 (2.2)	1 (1.1)
Multiple organ dysfunction syndrome	0	1 (2.2)	1 (1.1)
Injury, poisoning and procedural complications	0	1 (2.2)	1 (1.1)
Pelvic fracture	0	1 (2.2)	1 (1.1)
Psychiatric disorders	1 (2.2)	0	1 (1.1)
Suicidal ideation	1 (2.2)	0	1 (1.1)

List of ReMEDy1 Sites, Investigators, Study Coordinators, and Research Nurses

Site Name	Last Name	First Name	Study Role
Royal Melbourne Hospital	Campbell	Bruce	Principal Investigator
Royal Melbourne Hospital	Fisicchia	Laura	Primary Study Coordinator
Royal Melbourne Hospital	McDonald	Amy	Study Coordinator
Royal Melbourne Hospital	Jackson	David	Study Coordinator
Royal Melbourne Hospital	Alemseged	Fana	Sub-Investigator
Royal Melbourne Hospital	Yassi	Nawaf	Sub-Investigator
Royal Melbourne Hospital	Zhao	Henry	Sub-Investigator
Royal Melbourne Hospital	Parsons	Mark	Sub-Investigator
Royal Melbourne Hospital	Ng	Felix	Sub-Investigator
Royal Melbourne Hospital	Ng	Jo Lyn	Sub-Investigator
Royal Melbourne Hospital	William	Cameron	Sub-Investigator
Royal Melbourne Hospital	Smith	Kate	Study Coordinator
Royal Melbourne Hospital	Mcfadzen	Andrew	Study Coordinator
Royal Brisbane and Women's Hospital	Wong	Andrew	Principal Investigator
Royal Brisbane and Women's Hospital	Skinner	Genevieve	Primary Study Coordinator
Royal Brisbane and Women's Hospital	Muller	Claire	Sub-Investigator
Royal Brisbane and Women's Hospital	Roizman	Michael	Sub-Investigator
Fiona Stanley Hospital	Ghia	Darshan	Principal Investigator
Fiona Stanley Hospital	O'laughlin	Nicole	Primary Study Coordinator
Fiona Stanley Hospital	Edmonds	Gillian	Primary Study Coordinator
Fiona Stanley Hospital	Musuka	Tapuwa	Sub-Investigator
Royal Adelaide Hospital	Kleinig	Timothy	Principal Investigator
Royal Adelaide Hospital	Drew	Roy	Study Coordinator
Royal Adelaide Hospital	Cheong	Edmund	Sub-Investigator
Royal Adelaide Hospital	Cranefield	Jennifer	Primary Study Coordinator
Royal Adelaide Hospital	Mahadevan	Joshua	Sub-Investigator
Ballarat Hospital	Goss	Carmel	Study Coordinator
Ballarat Hospital	Sandford	Angela	Study Coordinator
Ballarat Hospital	Sahathevan	Ramesh	Principal Investigator
Ballarat Hospital	Kraemer	Thomas	Sub-Investigator
Ballarat Hospital	Hair	Casey	Study Coordinator
Box Hill Hospital	Dewey	Helen	Sub-Investigator
Box Hill Hospital	Thomas	Grace	Study Coordinator
Box Hill Hospital	Choi	Philip M.C.	Principal Investigator

Box Hill Hospital	Stephens	Karen	Research Nurse
Box Hill Hospital	Pachani	Naila	Study Coordinator
John Hunter Hospital	Esperon	Carlos Garcia	Principal Investigator
John Hunter Hospital	Spratt	Neil	Sub-Investigator
John Hunter Hospital	Belevski	Linda	Study Coordinator
John Hunter Hospital	Kaauwai	Lara	Study Coordinator
John Hunter Hospital	Russell-Dresser	Michelle	Study Coordinator
John Hunter Hospital	Wellings	Tom	Sub-Investigator
John Hunter Hospital	Kerr	Erin	Study Coordinator
John Hunter Hospital	Krishnamurthy	Venkatesh	Sub-Investigator
John Hunter Hospital	Royan	Angela	Study Coordinator
Liverpool Hospital	Hodgkinson	Suzanne	Sub-Investigator
Liverpool Hospital	Cordato	Dennis	Principal Investigator
Liverpool Hospital	Cheng	Qi Cheryl	Study Coordinator
Liverpool Hospital	Bhaskar	Sonu	Study Coordinator
Liverpool Hospital	Cappelen-Smith	Cecilia	Sub-Investigator
Liverpool Hospital	Calic	Zeljka	Sub-Investigator
Liverpool Hospital	McDougall	Alan	Sub-Investigator
Liverpool Hospital	Venkat	Abhay	Sub-Investigator
Liverpool Hospital	Blair	Christopher	Sub-Investigator
Liverpool Hospital	Edwards	Leon	Sub-Investigator
Liverpool Hospital	Phua	Chun	Sub-Investigator
Princess Alexandra Hospital	Bendall	Carol	Primary Study Coordinator
Princess Alexandra Hospital	Monaghan	Kathryn	Other
Princess Alexandra Hospital	Shah	Darshan	Principal Investigator
Princess Alexandra Hospital	Brown	Helen	Sub-Investigator
Princess Alexandra Hospital	Aikens	Philip	Sub-Investigator
Princess Alexandra Hospital	Sieg	Charmaine	Study Coordinator
Princess Alexandra Hospital	Atudawage	Chamudri Sagarika	Sub-Investigator
Princess Alexandra Hospital	Lau	Hung Tiong	Sub-Investigator
Sunshine Hospital (Western Health)	Wijeratne	Tissa	Principal Investigator
Sunshine Hospital (Western Health)	Celestino	Sherisse	Study Coordinator
Sunshine Hospital (Western Health)	Low	Essie	Study Coordinator
Sunshine Hospital (Western Health)	Mackey	Elizabeth	Study Coordinator
Sunshine Hospital (Western Health)	Tu	Hans	Sub-Investigator
Sunshine Hospital (Western Health)	Ng	Geraldine	Sub-Investigator
Sunshine Hospital (Western Health)	Bergqvist	Jennifer	Study Coordinator

Lismore Base Hospital	Boys	Janice	Primary Study Coordinator
Lismore Base Hospital	Moore	Stephen	Principal Investigator
Lismore Base Hospital	Hoffman	Kim	Study Coordinator
Lismore Base Hospital	Mishra	Amit	Sub-Investigator
Lismore Base Hospital	Frogley	Maree	Research Nurse
Lismore Base Hospital	Kozlowska	Jowita	Sub-Investigator
Lismore Base Hospital	Linsley-Brown	Clare	Research Nurse
The Alfred Hospital	Cloud	Geoffrey	Principal Investigator
The Alfred Hospital	Hand	Peter	Sub-Investigator
The Alfred Hospital	Zavala	Jorge	Sub-Investigator
The Alfred Hospital	Yip	Gary	Sub-Investigator
The Alfred Hospital	Moore	Andrea	Primary Study Coordinator
The Alfred Hospital	Zhang	Wenwen	Sub-Investigator
Royal North Shore Hospital	Krause	Martin	Principal Investigator
Royal North Shore Hospital	Day	Susan	Study Coordinator
Royal North Shore Hospital	Priglinger-Coorey	Miriam	Sub-Investigator
